# Accuracy of Narrow-Band Imaging International Colorectal Endoscopic Classification for Predicting the Histology of Colon Polyps by Experienced Endoscopists and Trainees

**DOI:** 10.5152/tjg.2023.22854

**Published:** 2023-08-01

**Authors:** Ramón Mena-Ramírez, Andres Macari-Jorge, Eva Juárez-Hernández, Misael Uribe, Iván López-Méndez

**Affiliations:** 1 Department of Gastroenterology and Obesity, Medica Sur Clinic & Foundation, Mexico City, Mexico; 2 Department of Pathological Anatomy, Medica Sur Clinic & Foundation, Mexico City, Mexico; 3 Department of Translational Research, Medica Sur Clinic & Foundation, Mexico City, Mexico; 4 Department of Endoscopy, Medica Sur Clinic & Foundation, Mexico City, Mexico

**Keywords:** Colonoscopy, colorectal neoplasms, narrow-band imaging, histology, polyps

## Abstract

**Background/Aims:**

Digital chromoendoscopy has proven to be useful in the histological prediction of premalignant lesions in the colon. The aim of the study was to describe the diagnostic performance of Narrow-Band Imaging International Colorectal Endoscopic Classification in the histological differentiation of colonic lesions, applied by expert endoscopists and trainees.

**Materials and Methods:**

Cross-sectional study that includes high-definition endoscopic images and histopathological reports of 94 patients over 50 years. Images were evaluated and classified as Narrow-Band Imaging International Colorectal Endoscopic 1, 2, or 3 by 2 experts and 2 trainee endoscopists, all of them blinded to histological results. Diagnostic accuracy for each Narrow-Band Imaging International Colorectal Endoscopic category was calculated for trainees and expert endoscopists. Intra-observer agreement was evaluated by means of Cohen’s kappa coefficient; meanwhile, inter-observer agreement was calculated by means of Fleiss’ kappa.

**Results:**

Evaluations performed by expert and trainee endoscopists showed a performance for Narrow-Band Imaging International Colorectal Endoscopic category 1: sensitivity 62%, specificity 85%, area under receiver operator characteristic 0.73; Narrow-Band Imaging International Colorectal Endoscopic category 2: sensitivity 61%, specificity 73%, area under receiver operator characteristic 0.66; and Narrow-Band Imaging International Colorectal Endoscopic category 3: sensitivity 88%, specificity 91%, area under receiver operator characteristic 0.86. The total agreement of the evaluations was 72.5%, with an inter-observer variability of *K* 0.60 (95% CI 0.52-0.74). When the diagnostic performance for non-dysplastic lesions and dysplastic lesions (Narrow-Band Imaging International Colorectal Endoscopic 1 vs 2 and 3) was compared, we observed an increase in sensitivity for differentiated adenomas (Narrow-Band Imaging International Colorectal Endoscopic 2).

**Conclusion:**

Narrow-Band Imaging International Colorectal Endoscopic Classification applied in the histological prediction of static images of colonic lesions has a good diagnostic performance for Narrow-Band Imaging International Colorectal Endoscopic category 3, as well as an acceptable performance for Narrow-Band Imaging International Colorectal Endoscopic category 1, with a moderate agreement among observers.

**Main Points**

- Digital chromoendoscopy has the purpose of increasing the number of colorectal lesions detected during colonoscopy and differentiating neoplastic and non-neoplastic polyps.
- The diagnostic performance of Narrow-Band Imaging International Colorectal Endoscopic (NICE) Classification is acceptable for NICE category 1 and good for NICE category 3 (according to the area under receiver operator characteristic values) for the detection of colonic lesions.
- No difference in the diagnostic performance of the classification was observed when applied by expert endoscopists and by trainees.
- Intra-observer and inter-observer agreements showed a moderate level of agreement between expert and trainee evaluators.

## Introduction

Colorectal cancer (CRC) represents 10% of all neoplasia and deaths related to cancer in the world.^1^ Incidence rates vary according to the geographic region, with developed countries showing the highest numbers. It is estimated that developing countries will show an increase in CRC incidence of up to 2.5 million cases by 2035, with a relative reduction in developed countries due to an improvement in screening programs, an increased use of colonoscopy, and lifestyle modifications.^2^

A colonic polyp is a cell cluster formed in the colon mucosa. There are 2 main categories: non-neoplastic (hyperplasic, inflammatory, and hamartomatous) and neoplastic (adenomas and serrated adenomas).^3^ Colon adenomas are pre-malignant epithelial tumors: the conventional adenoma (with its 3 possible histologies: tubular, tubulovillous, and villous), sessile serrated adenoma, and traditional serrated adenoma. The conventional tubular adenoma is the most frequent histological type of lesion and it corresponds to 65%-85% of resected lesions.^4,5^ The use of colonoscopy as a screening strategy for CRC has evolved since the 1990s when evidence emerged about its usefulness for reducing the incidence of CRC between 70% and 90%, and later on, its capability for reducing mortality up to 80%, specially deceases related to lesions located on the left side of the colon, and in a lower percentage in lesions located on the right side of the colon (due to factors related to colonoscopy quality and type of lessons). These benefits were mainly attributed to the resection of pre-malignant lesions (adenomas) with polypectomies.^6,7^

Traditional chromoendoscopy and digital chromoendoscopy are part of a set of techniques developed with the aim of increasing the number of lesions detected during colonoscopy as well as improving the capability of distinguishing between neoplastic and non-neoplastic polyps.^8^ Narrow-band imaging (NBI) is a modality that uses a light filter that only allows the passage of 1 wavelength (blue with 415 nm wavelength or green with 540 nm wavelength), which equals the hemoglobin absorption spectrum. The wavelength is absorbed by the blood vessels and produces an image in contrast with mucosa. Due to the size change and vascular pattern in the mucosa and submucosa when the tissue becomes pre-neoplastic or neoplastic, NBI offers the endoscopist the opportunity to predict the malignant potential of polypoid lesions in the colon. The possibility of distinguishing neoplastic from non-neoplastic lesions with the application of NBI has been studied and has given rise to classifications such as Narrow-Band Imaging International Colorectal Endoscopic (NICE) Classification and Japanese team of NBI experts in order to facilitate the identification of lesions and to predict their histology.^9-11^

The NICE Classification, described in 2012 by Hewet et al,^12^ takes the color of the lesion, the characteristics of vessels, and the surface pattern into account in order to categorize lesions as type 1 (corresponding to hyperplasic histology) and type 2 (corresponding to an adenoma). Type 3 lesions (corresponding to invasive adenocarcinomas)^13^ were added in subsequent studies. Studies evaluating the performance of the classification applied by endoscopists are not available in our environment; hence, the objective of this study was to describe the diagnostic performance of NICE Classification in the histological differentiation of colonic lesions applied by expert and trainee endoscopists.

## Materials And Methods

It is a cross-sectional, comparative study that includes high-definition endoscopic images and histopathological reports of patients over 50 years of age that came to the Medica Sur Hospital for a screening colonoscopy (early detection of colon cancer) during the period comprised between January 2017 and December 2019.

First, we identified histopathologic reports corresponding to polypoid colon lesions. Then retrospectively, we obtained high-definition endoscopic images of these reports and selected 2 high-quality images for each histopathologic report. Demographical and clinical data were collected as well.

Images with chromoendoscopy by NBI obtained with Olympus GIF-HQ 190 Evos Exera III endoscopes (made in Japan) of polypoid lesions were included. Lesions identification resulted in their endoscopic resection and subsequent analysis and report by the pathological anatomy department of the hospital. Low-quality images and those previously known by the evaluators were excluded. Images were randomized by means of a computerized survey application (Google formats) and access links were provided to 2 expert endoscopists with more than 5 years of experience in the use of NBI chromoendoscopy, as well as to 2 trainee endoscopists (fellow in endoscopy graduate doctors), all of them blinded to the histological results. Trainees and expert endoscopists were skilled in NBI and NICE Classification; however, they did not receive a specific training for this study.

With an estimated inter-observer agreement of 15%, according to previous studies^12^ with a 0.5 standard error and a confidence level of 95%, a sample size of 119 reports was estimated; data from a total sample of 130 reports were collected, adjusting for losses.

Images were evaluated in blocks of 30, with the option of categorizing each lesion into categories 1, 2, or 3 according to NICE Classification. In order to observe the agreement of the results, a set of second evaluations was performed 30 days after the initial evaluation, in which the same previously evaluated images were included but with a different randomization. Results were automatically downloaded to a Google spreadsheet and later recorded in another table for their analysis. Once the results were obtained, they were compared with histological results included in the corresponding reports. These 130 images represented a total of 520 observations among the 4 endoscopists.

The diagnostic accuracy, sensitivity (Sen), specificity (Spe), predictive values [positive predictive value (PPV) and negative predictive value (NPV)], and likelihood ratios for each category of NICE Classification were determined for each endoscopist in order to evaluate the primary objective. The intra-observer agreement was calculated by means of Cohen’s Kappa coefficient, and the inter-observer agreement was calculated by means of Fleiss’ Kappa with the Online Kappa Calculator software.^14^ Descriptive data are reported in percentages, means, or medians according to data distribution. The study was approved by the Médica Sur S.A.B. de C.V. Ethics and Research Committee with authorization number 2020-EXT-459 and in accordance with the provisions of the Declaration of Helsinki; the informed consent form was not required since this research involves minimal risk with a review of medical records for limited information, data are derived from clinically indicated procedures, and all data were anonymized to avoid breaches of confidentiality.

## Results

A total of 130 high-definition endoscopic images (520 observations) of polypoid colon lesions were included, corresponding to a total of 94 patients. The mean age was 62 ± 11 years; 67% (n = 63) of the images corresponded to male patients. As for the images included, 41% (n = 54) corresponded to lesions with histological reports of adenomas; 33% (n = 43) were hyperplasic polyps; and 25% (n = 33) were invasive adenocarcinomas. According to histopathological reports, 60% (n = 78) were polyps >10 mm.

The overall diagnostic accuracy of NICE for predicting colon polyp histology was 75.7%; 71.1% for NICE 1; 67.1% for NICE 2, and 89.9% for NICE 3. The diagnostic accuracy of NICE was 78% when performed by skilled endoscopists and 73.9% when performed by trainees. Results of diagnostic performances for each category of NICE Classification can be observed in Table 1.

Evaluations performed by experienced endoscopists revealed the following combined performance values: for NICE category 1: Sen 62%, Spe 85%, PPV 72%, NPV 79%, area under receiver operator characteristic (AUROC) 0.75; NICE category 2: Sen 61%, Spe 74%, PPV 64%, NPV 72%, AUROC 0.67; and NICE category 3: Sen 96%, Spe 91%, PPV 70%, NPV 99%, AUROC 0.84 (Table 1). The comparison between AUROCs did not show a significant difference.

The total concordance of evaluations was 75.38%, with an inter-observer agreement of *K* 0.63 (95% CI 0.52-0.74) for all the categories combined. The inter-observer agreement for each category was *K* 0.61 for NICE 1, *K* 0.49 for NICE 2, and *K* 0.79 for NICE 3. The intra-observer agreement of each expert evaluator was *K* 0.675 and *K* 0.677, while for that of the trainees was *K* 0.710 and *K* 0.772.

The evaluations carried out by trainee endoscopists revealed the following combined performance values: for NICE category 1: Sen 64%, Spe 79.5%, PPV 62%, NPV 76%, AUROC 0.71; NICE category 2: Sen 61%, Spe 73%, PPV 62%, NPV 71%, AUROC 0.66; and NICE category 3: Sen 81%, Spe 93%, PPV 8%1, NPV 94%, AUROC 0.85 (Table 1). The comparison between AUROCs did not show a significant difference. The total concordance of evaluations was 70%, with an inter-observer agreement of *K* 0.55 (95% CI 0.43-0.67) for all NICE categories, while the inter-observer agreement for each category was *K* 0.58 for NICE 1, *K* 0.41 for NICE 2, and *K* 0.67 for NICE 3. The intra-observer agreement for each trainee endoscopist was *K* 0.710 and *K* 0.772.

In an additional analysis, we compared the diagnostic performance for non-dysplastic lesions (NICE 1) versus NICE 2 and 3 categories. We observed an increase in sensitivity for differentiated adenomas (NICE 2) in trainees (78% and 79%) and expert endoscopists (75% and 82%) (Table 2).

All polyps were resected. Regarding the resection methods, hyperplasic lesions were resected in 60.5% (n = 26) with simple forceps, 13.5% (n = 6) with cold snare, 4.5% (n = 2) with hot snare, and no resection method was reported in 21.5% (n = 9) of the cases. As for adenomas, 56.6% (n = 30) were resected with cold snare, 28.3% (n = 15) with hot snare, 7.5% (n = 4) with forceps, and no resection method was reported for the remaining 7.5% (n = 4) of the cases. In the case of invasive lesions, 76% (n = 25) were only biopsied, 9% (n = 3) were resected with cold snare, 9% (n = 3) with hot snare, and no resection method was reported for 6% (n = 2) of the cases.

## Discussion

The results obtained in the study indicate that the diagnostic performance of NICE Classification applied to high-definition static images for the histologic prediction of polypoid lesions in the colon is acceptable for NICE category 1 and NICE category 3, according to the AUROC values obtained above 0.7 and 0.8, respectively.^15^ No significant difference in the diagnostic performance of the classification was observed when applied by expert endoscopists and trainees. Intra-observer and inter-observer agreements showed a moderate level of agreement between expert and trainee evaluators.

Multiple studies that have evaluated NICE Classification have reported different diagnostic performances in the histological prediction of colonic lesions. This variability has been explained by the different designs and scenarios in which the classification has been evaluated, as well as the level of experience of endoscopists that apply it and the endoscopy equipment used.^16-20^ In a recent study, Rees et al^20^ evaluated the usefulness of NICE Classification applied by 28 colonoscopists trained in NBI who rated 722 polyps <10mm in order to predict lesions with adenomatous histology. The results reported in the study showed Sen 83.4%, Spe 74.8%, NPV 64%, and PPV 89%. These results are similar to the diagnostic performance observed in our study. In a study carried out in Spain, Puig et al^16^ investigated the accuracy of NICE Classification for the identification of adenomatous polyps with submucosal invasion (NICE 3) by means of an assessment of data obtained by 58 endoscopists who evaluated 2123 lesions >10 mm. The results obtained showed Sen 58.4%, Spe 96%, PPV 41.6%, and NPV 98%, with AUROC 0.77, which is lower in comparison with the AUROC that we obtained in our study for NICE category 3 (AUROC 0.84). Regardless of the variability in the diagnostic performance of the classification, studies coincide in showing a good performance for the prediction of hyperplasic and invasive lesions, but not in the prediction of adenomatous lesions (NICE 2), where the results obtained both in our study and in other studies show lower performances, probably related to the morphologic variability of this kind of lesions and to the absence of macroscopic components that would allow for better predictions. Intra-observer and inter-observer agreements reported in the different studies mentioned here are consistently moderated, and so are those obtained in our results.

With regard to the results obtained in previous studies, in 2012 Hewet et al^12^ carried out a series of evaluations by expert endoscopists using static images of polyps. Lesions were classified as NICE 1 or NICE 2 and with a high (>90% of diagnostic certainty) or low (<90%) reliability level. Thus, the diagnostic accuracy of those lesions rated with high reliability was 98% (Sen 98%, Spe100%), while the diagnostic accuracy in lesions with low reliability was 95% (Sen 94%, Spe 97%) to distinguish neoplastic from non-neoplastic lesions. The inter-observer agreement was 0.87, and when it was limited to the lesions interpreted with high reliability, Kappa was 0.97. During a subsequent phase of the same studio, endoscopists were able to elaborate a “high reliability” prediction in 75% of the polyps evaluated in an assessment of the classification applied in a real-time colonoscopy scenario. These specialists achieved a diagnostic accuracy of 89%, with Sen 98%, Spe 69%, NPV 95%, and PPV 87%. The inter-observer Kappa obtained in this phase of the study was moderated (K 0.75). In another study carried out in 2013 by Hayashi et al,^13^ the diagnostic accuracy of the classification was evaluated including NICE category 3, reporting Sen 83%, Spe 70%, PPV 66.5%, and diagnostic accuracy 75.6%, with a substantial agreement among evaluators (K 0.70). These results are similar to those obtained in our study.

It has been suggested that the endoscopic determination of colorectal polyps histology can potentially prevent unnecessary polypectomies or allow for a strategy in which all polyps are resected and a surveillance system based on “optical biopsy” is applied, which could substantially reduce costs related to the histopathological analysis of resected lesions.^21^ The recommendation of the American Society for Gastrointestinal Endoscopy (ASGE) includes that any “resect and discard” strategy for polyps must have >90% concordance with recommendations based on histological results, as well as the need of >90% of NPV for adenomatous histology if the option of resecting tiny polyps in the rectosigmoid is not desired.^22^ The NICE Classification does not seem to comply consistently with enough requirements to allow for a follow-up strategy based on “optic biopsy” that satisfies the criteria proposed by the ASGE, according to the results obtained in our study and those reported in different studies. The best performances of this classification reported in the NICE category 3 might allow the selection of the resection method that is appropriate for a lesion, because if the endoscopist observes data of submucosal invasion (size >40-60 mm, ulceration, excavation or depression, irregular edges, laterally extended lesions) during the evaluation, a resection of the polyp is necessary by means of an endoscopic submucosal dissection, due to the high risk of incomplete resection if the lesion is treated with other methods.^23^

The limitations of our study are those related to its observational design and to the fact that it was carried out in a single third-level center. The size of polyps was not limited for their inclusion in the study and it has been observed that size could be under or over-estimated for the endoscopic forceps size^24^; however, the aim of this study was not related to polyp size; evaluations were performed with high-definition static images. The strengths of our study are that we included polyps without limiting their size; thus, it is possible to reflect a scenario that is reproducible in other environments if global evaluations are carried out; also, the randomized design and the evaluation in 2 events, which proved reproducibility of the results in evaluators, as well as the number of observations accumulated (520 evaluations). As far as we know, and according to the literature review that we carried out, this is the only study with endoscopic images and histological results in our location. It is desirable for future works to carry out the evaluations with real-time images in a prospective way.

According to our results, the performance of the NICE scale is very good in excluding non-dysplastic lesions and in highly suspected lesions (NICE 1 and 3); nonetheless, in NICE 2, where the possibilities are superimposed, the performance is lower. With this study, we could evaluate the diagnostic accuracy for polypoid lesions in a daily clinical setting with trainee and expert endoscopists skilled in NICE Classifications; nonetheless, evaluating accuracy variations before and after a specific and detailed training program could be interesting.

In conclusion, the NICE Classification applied by expert and trainee endoscopists for the histological prediction of static images of colon lesions has a good diagnostic performance for NICE category 3 and NICE category 1.

## Figures and Tables

**Table 1.** Diagnostic Accuracy of NICE Classification by Experts and Trainees

	NICE 1						
	Sen (95% CI)	Spe (95% CI)	PPV	NPV	PLR	NLR	AUROC
Expert 1	58% (0.43-0.73)	84% (0.76-0.92)	72%	75%	3.60	0.50	0.734
Expert 2	66% (0.52-0.80)	86% (0.79-0.93)	72%	82%	4.70	0.40	0.769
Trainee 1	61% (0.46-0.76)	84% (0.76-0.92)	70%	78%	3.81	0.46	0.740
Trainee 2	66% (0.52-0.80)	64% (0.54-0.74)	53%	74%	1.83	0.53	0.698
Combined expert	62% (0.46-0.77)	85% (0.74-0.96)	72%	79%	4.14	0.45	0.75
Combined trainee	64% (0.50-0.78)	79.5% (0.67-0.92)	62%	76%	3.12	0.45	0.71
	NICE 2						
Expert 1	58% (0.43-0.73)	71% (0.61-0.81)	59%	70%	2.00	0.59	0.645
Expert 2	65% (0.52-0.78)	77% (0.68-0.86)	69%	74%	2.80	0.45	0.711
Trainee 1	64% (0.51-0.77)	71% (0.61-0.81)	54%	79%	2.21	0.50	0.633
Trainee 2	57% (0.44-0.70)	74% (0.64-0.84)	69%	63%	2.10	0.58	0.658
Combined expert	61% (0.48-0.74)	74% (0.62-0.86)	64%	72%	2.35	0.53	0.67
Combined trainee	61% (0.48-0.74)	73% (0.61-0.85)	62%	71%	2.26	0.53	0.66
	NICE 3						
Expert 1	100%	90% (0.83-100)	67%	100%	10	0	0.833
Expert 2	92% (0.84-0.96)	91% (0.86-0.96)	73%	98%	10.22	0.09	0.853
Trainee 1	78% (0.62-0.94)	95% (0.91-0.99)	85%	92%	15.60	0.23	0.883
Trainee 2	83% (0.69-0.97)	91% (0.86-0.96)	76%	94%	9.22	0.19	0.833
Combined expert	96% (0.89-100)	91% (0.81-100)	70%	99%	11	0.04	0.84
Combined trainee	81% (0.68-0.94)	93% (0.84-100)	81%	94%	12	0.20	0.85

AUROC, area under receiver operator characteristic; NLR, negative likelihood ratio; NPV, negative predictive value; PLR, positive likelihood ratio; PPV, positive predictive value; Sen, sensitivity; Spe, specificity.

**Table 2.** Diagnostic Accuracy of NICE Classification in Predicting Adenomatous Versus Non-Adenomatous Histology

	Sen (95% CI)	Spe (95% CI)	PPV	NPV	PLR	NLR	AUROC
Expert 1	75% (0.65-0.84)	72% (0.59-0.85)	54%	57%	2.68	0.35	0.715
Expert 2	82% (0.74-0.90)	72% (0.59-0.85)	86%	76%	2.93	0.25	0.757
Trainee 1	78% (0.69-0.86)	70% (0.59-0.83)	84%	61%	2.60	0.31	0.726
Trainee 2	79% (0.71-0.87)	66% (0.52-0.80)	86%	53%	2.32	0.32	0.723

AUROC, area under receiver operator characteristic; NLR, negative likelihood ratio; NPV, negative predictive value; PLR, positive likelihood ratio; PPV, positive predictive value; Sen, sensitivity; Spe, specificity.
